# Differential Role of Smad2 and Smad3 in the Acquisition of an Endovascular Trophoblast-Like Phenotype and Preeclampsia

**DOI:** 10.3389/fendo.2020.00436

**Published:** 2020-07-08

**Authors:** Jelena Brkić, Caroline Dunk, Yanan Shan, Jacob Anderson O'Brien, Phetcharawan Lye, Sheza Qayyum, Peifeng Yang, Stephen G. Matthews, Stephen J. Lye, Chun Peng

**Affiliations:** ^1^Department of Biology, York University, Toronto, ON, Canada; ^2^Lunenfeld Tanenbaum Research Institute, Mount Sinai Hospital, Toronto, ON, Canada; ^3^Department of Physiology, University of Toronto, Toronto, ON, Canada; ^4^Centre for Research in Biomolecular Interactions, York University, Toronto, ON, Canada

**Keywords:** Smad2, Smad3, preeclampsia, placenta, endovascular trophoblasts

## Abstract

During placental development, cytotrophoblast progenitor cells differentiate into the syncytiotrophoblast and invasive extravillous trophoblasts (EVTs). Some EVTs further differentiate into endovascular trophoblasts (enEVTs) which exhibit endothelial-like properties. Abnormal placental development, including insufficient enEVT-mediated remodeling of the uterine spiral arteries, is thought to be a precipitating factor in the onset of preeclampsia (PE), a pregnancy-related hypertensive disorder. Several members of the transforming growth factor-β (TGF-β) superfamily, such as TGF-βs, Nodal, and Activin have been reported to either promote or inhibit the invasive EVT pathway. These ligands signal through serine/threonine receptor complexes to activate downstream signaling mediators, Smad2 and Smad3. In this study, we determined Smad2 and Smad3 expression pattern in placenta and their effects on trophoblast invasion and differentiation. Total Smad2/3 levels were relatively constant across gestation while the ratio of active phosphorylated forms to their total levels varied with gestational stages, with a higher pSmad2/total Smad2 in later gestation and a higher pSmad3/total Smad3 in early gestation. Immunofluorescent staining revealed that pSmad3 was localized in nuclei of EVTs in anchoring villi. On the other hand, pSmad2 was mostly absent in this invasive EVT population. In addition, pSmad3/total Smad3, but not pSmad2/total Smad2, was significantly lower in both early onset and late onset PE cases, as compared to gestational age-matched controls. Functional studies carried out using a first trimester trophoblast cell line, HTR-8/SVneo, and first trimester human placental explants showed that Smad2 and Smad3 had differential roles in the invasive pathway. Specifically, siRNA-mediated knockdown of Smad2 resulted in an increase in trophoblast invasion and an upregulation of mRNA levels of enEVT markers while the opposite was observed with Smad3 knockdown. In addition, Smad2 siRNA accelerated the EVT outgrowth in first trimester placental explants while the Smad3 siRNA reduced the outgrowth of EVTs when compared to the control. Furthermore, knockdown of Smad2 enhanced, whereas overexpression of Smad2 suppressed, the ability of trophoblasts to form endothelial-like networks. Conversely, Smad3 had opposite effects as Smad2 on network formation. These findings suggest that Smad2 and Smad3 have opposite functions in the acquisition of an enEVT-like phenotype and defects in Smad3 activation are associated with PE.

## Introduction

Serving as the interface between the fetal and maternal environments, the placenta plays a critical role in maintaining and protecting the developing fetus throughout pregnancy. Following fertilization, the polarized outer cells of the blastocyst differentiate into cells of the trophoblast lineage. The cytotrophoblast (CTB) progenitor cells, located at the basement membrane of the placental villi, differentiate in two general pathways (1, 2). In the fusion pathway, CTBs form the multinucleated syncytium that lines the developing primary placental villi. In the invasive pathway, proliferative CTBs at the tips of the early chorionic villi form anchoring columns that attach the developing placenta to the decidual stroma and generate the two invasive populations of extravillous cytotrophoblasts (EVTs), interstitial EVTs (iEVTs), and endovascular trophoblasts (enEVTs). iEVTs invade into the decidual and myometrial stroma while enEVTs invade the lumen of the maternal uterine spiral arteries where they replace the endothelial cells and acquire endothelial-like characteristics. This process transforms the spiral arteries into wide bore vessels which provide the steady, low-velocity blood flow to the placenta in order to meet the requirements of the growing fetus (3). Recent studies suggest that there are more EVT subtypes, including endoglandular trophoblasts, endovenous trophoblasts, endoarterial trophoblasts, and endolymphatic trophoblasts, which likely arise during the development of the trophoblast shell during early implantation (4, 5). Disruption of EVT differentiation, invasion, and spiral artery remodeling is thought to be associated with several pregnancy-related disorders, such as intrauterine growth restriction and preeclampsia (PE) (5–7).

PE is a multifactorial disorder manifested during pregnancy, posing a major risk to both the mother and fetus. It is typically diagnosed after 20 weeks of gestation as a maternal syndrome defined by the *de novo* sudden onset of hypertension, together with proteinuria or evidence of a systemic disease (8). Shallow EVT invasion into the decidua and failure of uterine spiral artery remodeling have been suggested to be major contributing factors of PE (6, 9). However, more recent studies suggest that defective EVT functions are more likely to be associated with only the more severe form of early onset PE (<34 weeks) while late onset PE is likely due to maternal factors (10, 11). EVT invasion and spiral artery remodeling are tightly regulated by many signaling networks, including the transforming growth factor (TGF)-β pathway (12, 13).

The TGF-β superfamily is a large group of growth factors involved in the regulation of many developmental and physiological processes. This superfamily of signaling molecules includes the TGF-βs, Activins, Nodal, and bone morphogenetic proteins (BMPs) (14). The canonical TGF-β signaling pathway involves binding to the type II and type I serine/threonine receptors and subsequent activation of the downstream receptor-regulated Smads (R-Smads). Two of the R-Smads, Smad2, and Smad3, are known to be activated by TGF-β, Activin, and Nodal (14, 15). Once R-Smads are phosphorylated by type I receptors, they form a complex with the common Smad4 and translocate to the nucleus where they regulate gene transcription (15). While Smad2 and Smad3 share sequence similarities, Smad2 differs from Smad3 mainly in the N-terminal MH1 domain where an additional sequence insertion perturbs its ability for direct DNA binding (16, 17). Targeted disruption of *Smad2* and *Smad3* genes revealed that they have both distinct and overlapping functions (18–22).

The TGF-β pathway is involved in many aspects of placental development. Ligands, receptors, and Smads are expressed in trophoblasts and many of them are dysregulated in PE (12, 23–25). Although TGF-β, Activin, and Nodal activate Smad2 and Smad3, they have different functions in regulating trophoblast differentiation and invasion. Activin A has been reported to promote EVT differentiation and invasion (26, 27). On the other hand, TGFβ-1 and−3 and Nodal inhibit this pathway (12). For example, all three TGFβ isoforms have been reported to inhibit trophoblast invasion and/or EVT outgrowth (28–33). Similarly, Nodal has also been found to inhibit migration, invasion, and placenta EVT outgrowth (29, 34–37). However, the pro-migratory and pro-invasive effects of TGFβ (38) and Nodal (39) in trophoblasts have also been observed. The mechanisms responsible for different TGF-β signaling responses are still not clear.

Recently, we reported that miR-218-5p promotes trophoblast differentiation into enEVTs and spiral artery remodeling through the suppression of TGFβ2 (40). We also found that Smad signaling is down-regulated in miR-218-5p overexpressing cells (40). In this study, we investigated the role of Smad2 and Smad3 in EVT invasion and differentiation to the enEVT phenotype, as well as their potential involvement in PE. We demonstrate that Smad2 inhibits while Smad3 promotes cell invasion, expression of enEVT markers, formation of endothelial-like networks, and EVT outgrowth in first trimester placental explants and that placentas from patients diagnosed with PE exhibit strong down-regulation of pSmad3.

## Materials and Methods

### Patients and Placental Tissue Collection

Fresh and frozen human placenta tissues were collected through the BioBank program at the Research Centre for Women's and Infants' Health (WCWIH) at Mount Sinai Hospital. This study was carried out in accordance with the recommendations of the Mount Sinai Hospital Research Ethics Board, Sinai Health System. The protocol was approved by the Mount Sinai Hospital Research Ethics Board, Sinai Health System (REB# 02-0061A and 11-0227-E). All subjects gave written informed consent in accordance with the Declaration of Helsinki. All research using human tissues was performed in a Class II certified laboratory by qualified staff trained in biological and chemical safety protocols, and in accordance with Health Canada guidelines and regulations. To determine the level of total and phosphorylated Smad2 and Smad3 across gestation, 13 placentas from 6 to 20 weeks collected from elective surgical terminations of pregnancies with no known pathology were used and 10 placentas from 25 to 40 weeks from preterm laboring and term elective deliveries were analyzed. To examine pSmad2 and pSmad3 in the EVT population, anchoring columns from 3 placentas between 10 and 14 weeks of gestation were used. In addition, to assess if there is dysregulation of Smad2/3 in PE, we used 3 placentas from early onset PE patients delivered between 28–31 weeks and 3 placentas from 36.6 to 39 weeks and 3 gestational age-matched controls for each PE group. PE was diagnosed when previously normotensive patients showed systolic blood pressure of >140 mm Hg or diastolic blood pressure of >90 mm Hg on at least 2 occasions, accompanied by positive dipstick tests for proteinuria (>1+) after 20 weeks of gestation or elevated liver enzymes. Villous tissues were collected from the maternal side penetrating to the fetal side. Every tube from the biobank has pieces from all depths of the placenta and from each quadrant sampled thus ensuring a representative placental sample. Clinical characteristics of the PE patients and controls are listed in Table 1. Finally, 6 placentas from 6 to 7.5 weeks were used in villous explant culture to assess EVT outgrowth.

**Table 1:** Clinical characteristics of preeclamptic patients and their gestational age-matched controls.

**Demographic**	**Preterm control (*n* = 3)**	**Early onset PE (*n* = 3)**	**Term control (*n* = 3)**	**Term PE (*n* = 3)**
**Age (years)**				
Mean	28.33	34	32.33	33.66
Range	23–35	32–41	28–37	21–43
**Parity**				
Nullipara	3	3	1	2
Multipara	0	0	2	1
**Gestational age at delivery**				
Mean (weeks)	31.43	29.90	38.87	37.76
Range	29.0–33.7	28.6–31.0	38.4–39.6	36.6–39.3
Cesarean delivery	1	3	3	2
Max sBP	115 ± 5.51	172 ± 6.11^**^	120 ± 1.53	158 ± 4.62^**^
Max dBP	74.67 ± 7.06	109.67 ± 6.89^*^	79.67 ± 1.20	101.33 ± 0.88^***^
Proteinuria (dip stick)	Negative or NA	3–4	NA	1–4
AST (u/L)	NA	43–45	NA	14–38
ALT (u/L)	NA	34–100	NA	10–20
Creatinine (mg/dL)	NA	50–70	NA	48–56
Chorioamnionitis	2	0	0	0
**Umbilical artery Doppler**				
>1.6 or notching	No	Yes	NA	NA
Birthweight (g)	1830 ± 321.9	1280 ± 230.9	3706.7 ± 49.1	2903.3 ± 278.7^*^
**Birthweight percentile**				
>10 and ≤ 50	0	3	3	1
>50 and ≤ 90	3	0	0	2
**Gender**				
Male	0	1	1	2
Female	3	2	2	1

* P < 0.05,

** P < 0.01; and

*** *P < 0.001 vs. corresponding control. NA, not available*.

### Protein Extraction and Immunoblot Analysis

Snap frozen tissues were lysed in five volumes of HEPES lysis buffer (10 mM HEPES, 10 mM NaCl, 0.1 mM EDTA, 0.1 mM EGTA, 1.0 mM DTT, 0.1% NP-40, pH 7.9) with a Pierce Protease and Phosphatase inhibitor (Thermo Fisher Scientific) and homogenized for 30 s twice while kept on ice. Proteins were quantified using the Pierce BCA Protein Assay Kit (Thermo Fisher). Equal amounts of protein were separated by SDS-polyacrylamide gel electrophoresis and transferred to a polyvinylidene difluoride membrane (Immobilon-P, Millipore Corp.). Membranes were blocked in 5% blocking buffer (5% skim milk in Tris-buffered Saline and Tween-20) for 1 h at room temperature, then incubated overnight with a primary antibody at 4°C (Table 2). Membranes were subsequently probed using horseradish peroxidase–conjugated secondary antibody (1:5,000) at room temperature for 2 h. Signals were detected using the ECL Plus Kit (Amersham Biosciences).

**Table 2:** Primary antibodies and staining reagents.

**Antibody**	**Company**	**Cat No**.	**Application**	**Species**	**Dilution**	**Diluent**
pSmad2	Cell Signaling	3101S	WB	Rabbit	1:500	5% Milk-TBST
pSmad3	Cell Signaling	9520S	WB	Rabbit	1:500	5% BSA-TBST
Smad3	Invitrogen	511500	WB	Rabbit	1:150	5% Milk-TBST
Smad2/3	Cell Signaling	3102S	WB	Rabbit	1:1,000	5% BSA-TBST
GAPDH	Santa Cruz	sc-47724	WB	Mouse	1:5,000	5% Milk-TBST
pSmad2	Thermo Fisher	44-244G	IF	Rabbit	1:500	PBS
pSmad3	Abcam	ab52903	IF	Rabbit	1:25	PBS
HLA-G	ExBio	11-499	IF	Mouse	1:300	PBS
Calcein AM	Corning	354217	Cell staining	N/A	1 μM	Serum-free media

### Immunofluorescent Staining

Dual immunofluorescent analysis was performed to detect the co-localization of pSmad2 or pSmad3 with HLA-G in anchoring columns of healthy first trimester placentas. Antigen retrieval for all antibodies used was performed by boiling slides in 10 mM sodium citrate. To reduce autofluorescence and background, tissues were incubated with 0.1% Sudan Black solution in 70% Ethanol for 2 min. A blocking buffer of 10% goat serum and 2% rabbit serum (Dako, Burlington, ON, Canada) was used in a humidified chamber for 1 h at room temperature followed by an overnight incubation at 4°C with primary antibody (Table 2). Incubation with mouse or rabbit IgG (1:100, Dako) in place of primary antibody served as a negative control. Excess antibody was washed off and subsequent incubations with secondary antibodies anti-mouse A488 (for HLA-G; 1:300) or biotinylated anti-rabbit (for pSmads; 1:300) followed by Streptavidin A546 (1:1,000) and DAPI (1:1,000) were performed for 1 h each at room temperature. Photographs were taken with a Zeiss LSM 700 Confocal microscope equipped with ZEN Software.

### Cell Culture

An immortalized cell line derived from human first trimester placenta, HTR-8/SVneo, was obtained from Dr. C. Graham (Queen's University, Kingston, ON, Canada) (41). The cell line was authenticated by IDEXX BioResearch in December 2017. Another trophoblast cell line generated from first trimester human placenta, Swan 71 (42), was a gift from Dr. G. Mor (Yale University, New Haven, CT, USA). Both cell lines were maintained in RPMI 1640 supplemented with 10% Fetal Bovine Serum (FBS), in an atmosphere of 5% CO_2_ at 37°C. All reagents were purchased from Thermo Fisher Scientific (Mississauga, Canada).

### Transient Transfections

Transient transfection of siRNA oligomers (100 nM) was carried out using Lipofectamine RNAiMax (Invitrogen, Thermo Fisher). All plasmid transfections (1 μg) were carried out using Lipofectamine 2000 (Invitrogen). A modified protocol was used to optimize transfection efficiency and cell survival. Transfections were carried out in 6-well plates on 70% confluent cultures. For each transfection reaction, 2 μl of Lipofectamine reagent was used and incubated with nucleotides for 15 min at room temperature in Opti-MEM media (GIBCO, Thermo Fisher). Transfections were carried out for 5 h and cells were recovered in 10% FBS containing media for 16 h. Cells used for marker analysis were recovered for an additional 24 h in serum-free media. siSmad2, siSmad3, and non-targeting controls were purchased from GenePharma Co. (Shanghai, China). The sequences of siRNAs are listed in Table 3. The Smad2 and Smad3 wild type constructs were obtained as described previously (23).

**Table 3:** Sequences of primers and oligomers.

**Name**	**Sequence: 5′ 3′**	**NCBI BLAST**
Cyc1	**F:** CAGATAGCCAAGGATGTGTG	Cytochrome c1
	**R:** CATCATCAACATCTTGAGCC	
VE-cad	**F:** GCCAGTTCTTCCGAGTCACA	Cadherin 5
	**R:** TTTCCTGTGGGGGTTCCAGT	
MMP1	**F:** GTCTCACAGCTTCCCAGCGA	Matrix Metallopeptidase 1
	**R:** ATGGCATGGTCCACATCTGC	
IL1b	**F:** AATCTGTACCTGTCCTGCGTGTT	Interleukin 1 beta
	**R:** TGGGTAATTTTTGGGATCTACACTCT	
IL8	**F:** CAGAGACAGCAGAGCACACA	C-X-C motif chemokine ligand 8
	**R:** GGCAAAACTGCACCTTCACA	
ECSCR	**F:** ACAACTCCCAGCCCACAATG	Endothelial Cell-Specific Chemotaxis Regulator
	**R:** GTGGTCAGACTTAGACCGCC	
siSmad2	**Sense:** GUCCCAUGAAAAGACUUAAtt	SMAD family member 2
	**Anti-sense:** UUAAGUCUUUUCAUGGGACtt	
siSmad3	**Sense:** CUGUGUGAGUUCGCCUUCAtt	SMAD family member 3
	**Anti-sense:** UGAAGGCGAACUCACACAGtt	
Control	UUCUCCGAACGUGUCACGUtt	No match
	ACGUGACACGUUCGGAGAAtt	

### RNA Extraction, Reverse Transcription, and qRT-PCR

Total RNA was extracted from cultured cells using TRIzol reagent (Invitrogen) as per manufacturer's protocol. Reverse transcription was performed on 1.5 μg total RNA with M-MuLV Reverse Transcriptase (New England Biolabs). Quantitative Real Time PCR was carried out using EvaGreen qPCR Master Mix (Applied Biological Materials Inc., Richmond, Canada), following the manufacturer's suggested protocol. All target genes were normalized to cytochrome c1 (Cyc1). The relative mRNA level was calculated using the 2^−ΔΔct^ method. All primers used in this study are listed in Table 3.

### Transwell Invasion Assay

Transwell inserts with 8 μm pores (Corning Inc.) were coated with Celtrex Reduced Growth Factor BM extract-PathClear (1:100 in Serum Free media, Trevigen) and allowed to polymerize overnight at 37°C. Cells were gently removed from culture plates using Accutase (Innovative Cell Technologies), and seeded at a density of 20,000 cells per well in serum-free RMPI1640 media. As a chemotactic agent, 10% serum containing medium was seeded on the outside of the transwells. After 24 h, membranes were fixed for 2 min in 100% methanol and stained using Harleco Hemacolor Staining Kit (EMD Chemicals). Invaded cells were counted with ImageJ (43).

### First-Trimester Human Placental Explant Culture

Explant cultures were performed as previously described (34, 40). Briefly, villous explants with potential EVT columns were carefully dissected and positioned on Transwell inserts (Millipore, Billerica, MA) pre-coated with 200 μL of undiluted phenol red-free Matrigel (BD Biosciences, Bedford, MA). Explants were left overnight to attach to the Matrigel, at 37°C with 3% O_2_ and 5% CO_2_, before adding serum-free DMEM-F12 medium supplemented with 100 U/mL of penicillin, 100 U/mL of streptomycin, 100 μg/mL Normacin™. After 2 days of culture, villous tips were examined under the dissecting microscope for successful EVT outgrowths. All successful explants were selected for treatment with 200 nM oligomers (Table 3). Explants were photographed immediately after adding treatment, and subsequently at 48 h, using a Leica DFC400 camera attached to a dissecting microscope. ImageJ was used to measure the area of EVT outgrowth. Specifically, total outgrowth area was calculated by subtracting the area at the end point with the initial area upon treatment. Each experiment was designed with a minimum of four replicates, and was repeated on three different placentas.

### Endothelial-Like Network Formation Assay

Network formation assays were carried out as previously reported (40). Briefly, HTR-8/SVneo cells were seeded onto 96-well plates coated with Celtrex Reduced Growth Factor BM extract-PathClear (Trevigen) at the density of 25,000 cells per well. After 16–20 h of culture, cells were stained with 1 μM Calcein AM (Corning) for 15 min, and pictures were taken at 20X magnification on a fluorescence microscope. Total network length was quantified in ImageJ using the NeuronJ plugin (44).

### Statistical Analysis

Statistical analysis was performed using Graphpad Prism 7 (*p* < 0.05, 95% CI). Unpaired, two-tailed, Student *t*-test was used for comparison between two groups. For experiments involving more than two groups, one-way ANOVA was performed, followed by a Dunnett's multiple comparisons test.

## Results

### The Active Forms of Smad2 and Smad3 Display Differential Patterns in the Placenta

**Figure 1:**
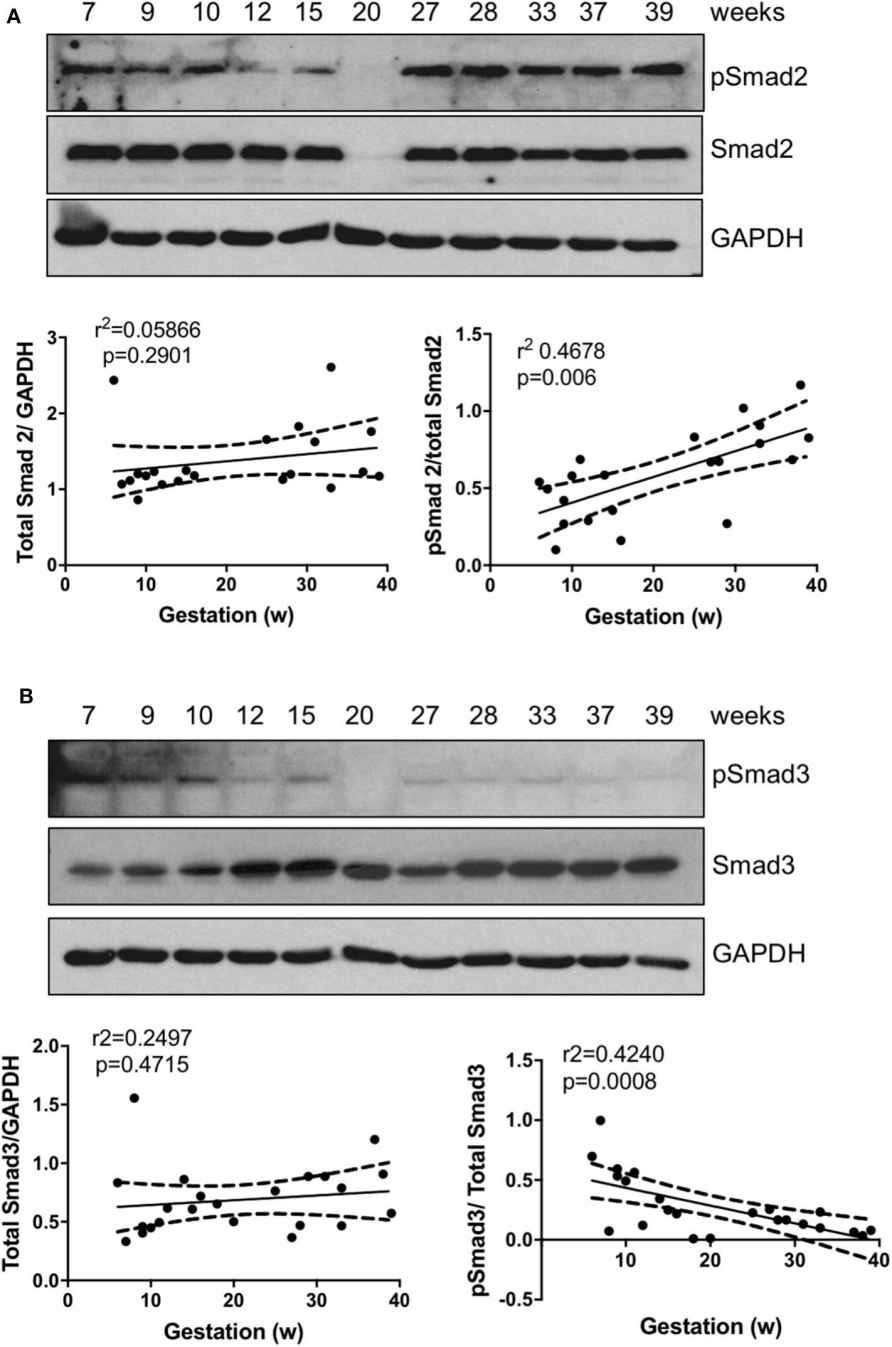
Smad2 and Smad3 expression in placentas across gestation. Healthy placentas from 6 to 39 weeks of pregnancy were assessed for total and phosphor-(p) Smad2 **(A)** and Smad3 **(B)** levels using Western Blotting. Representative blots and linear regression graphs depicting the relationship between their levels and gestational stage. The number on the top of each lane indicates gestational age of the sample.

To explore the roles of Smad2 and Smad3 in placenta development, we first assessed their expression pattern across gestation. Linear regression revealed that there was no significant association between either total Smad2 or total Smad3 levels with gestational age; however, the ratio of active form of Smad2, pSmad2, to the total Smad2 was positively associated with gestational age (Figure 1A) while pSmad3/total Smad3 showed a negative correlation with the increase in gestation age (Figure 1B).

**Figure 2:**
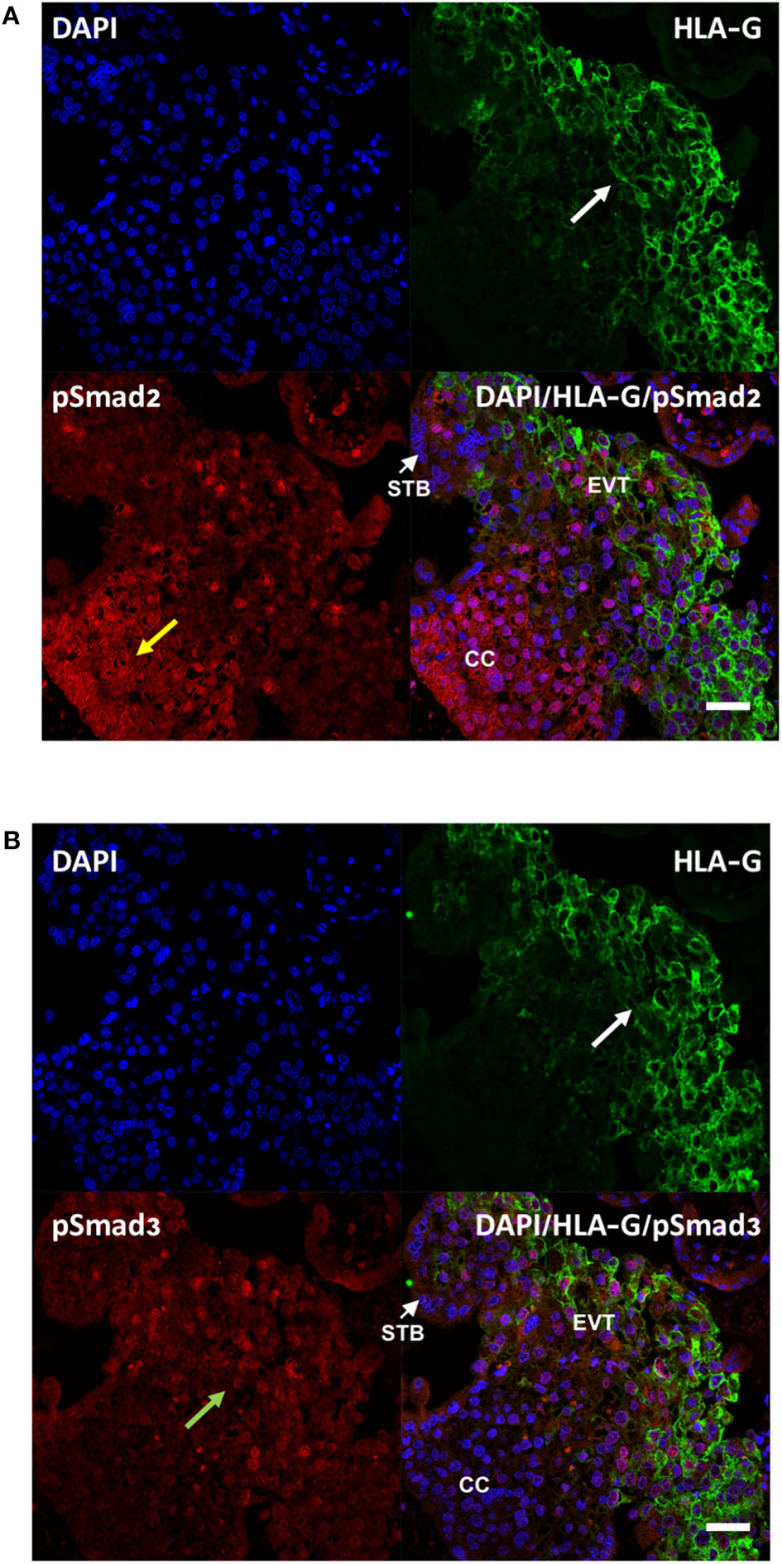
Differential cellular localization of the active forms of Smad2 **(A)** and Smad3 **(B)** in anchoring villi. Immunofluorescence staining was performed on 10–14 week placentas (*n* = 3) using antibodies against pSmad2 or pSmad3, as well as HLA-G to identify EVTs. Nuclei were stained with DAPI. Representative pictures from a 12-week placenta are shown. Nuclear staining for pSmad2 was more prominent in HLA-G negative cells in the proximal column cells (CC, yellow arrow), while pSmad3 (green arrow) was present mostly in nuclei of cells in the distal region where HLA-G positive EVT cells are present (white arrow). Scale bar = 40 μm. STB, syncytiotrophoblast.

To further examine the activation of Smad2 and Smad3 in the placenta, especially in EVTs, double immunofluorescence microscopy was performed on first trimester placenta tissues to detect the co-localization of pSmad2 or pSmad3 with the EVT marker, HLA-G. As shown in Figure 2, strong nuclear pSmad2 signals were mostly present in the HLA-G negative proximal cell column of CTBs. In the distal HLA-G positive differentiated EVT population, only a few cells displayed nuclear pSmad2 signals (Figure 2A). In contrast, pSmad3 signals were stronger in the EVT region but weaker in the cell column (Figure 2B).

### Phosphor-Smad3 Is Strongly Downregulated in Preeclamptic Placenta

**Figure 3:**
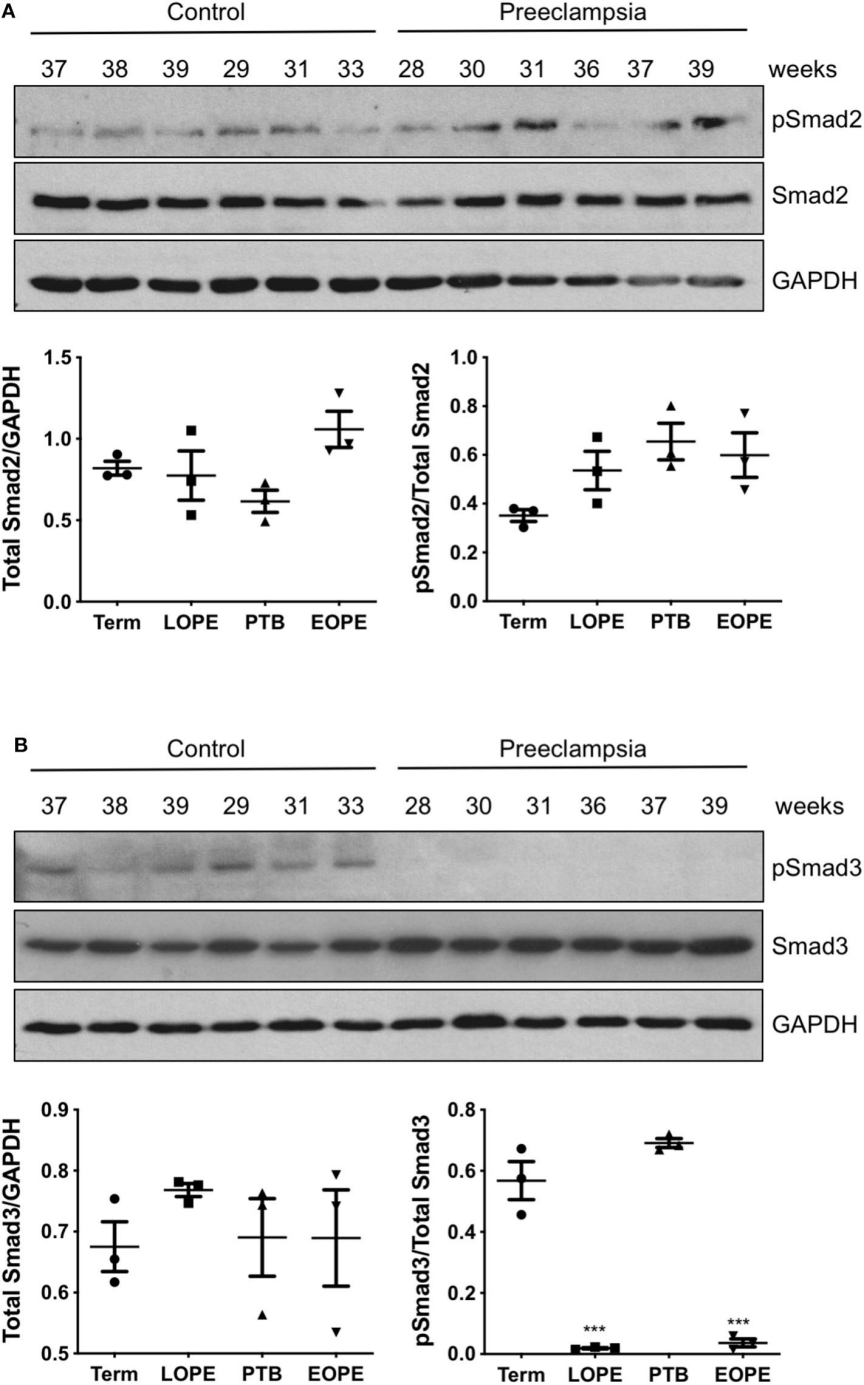
Active Smad3 is downregulated in preeclamptic placentas. Placentas from PE patients and gestational age-matched controls were assessed for total and phosphorylated (p) Smad2 **(A)** and Smad3 **(B)** by Western Blotting. Total Smad2 and Smad3 levels, after normalizing to their respective GAPDH signal, showed no significant difference between the PE and control placentas. The ratio of pSmad2/total Smad2 was also not significantly different between the PE and the corresponding control. However, pSmad3/total Smad3 was significantly decreased (***p < 0.001) in PE placentas relative to their gestational age-matched controls (*n* = 3). The number on the top of each lane indicates gestational age of the sample. PTB, preterm birth; EOPT, early onset preeclampsia; Term, healthy term placentas; LOPE, late onset preeclampsia.

Total and phosphorylated forms of Smad2 and Smad3 were also measured in placentas obtained from PE patients and compared with gestational age-matched controls. In both early onset and late onset PE, total Smad2 and pSmad2/total Smad2 ratio were not different from their gestational age-matched controls (Figure 3A). Total Smad3 levels were also similar between the control and PE placentas; however, strong down-regulation of pSmad3/total Smad3 was observed in both groups of PE cases, as compared to preterm birth or healthy term placentas (Figure 3B).

### Smad2 Induces, While Smad3 Inhibits, Acquisition of an enEVT-Like Phenotype

**Figure 4:**
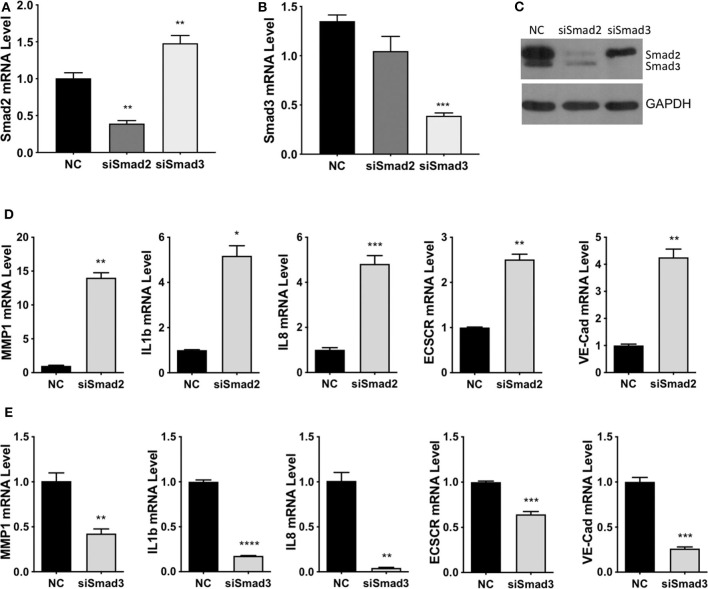
Smad2 and Smad3 exert opposite effects on the expression of key genes involved in enEVT differentiation. HTR-8/SVneo cells were transfected with a siRNA targeting Smad2 (siSmad2) or Smad3 (siSmad3) or a non-targeting control (NC). Total RNA or protein was extracted. **(A)** Validation of Smad2 siRNA (siSmad2) by qPCR. **(B)** Validation of siSmad3 using qPCR. **(C)** Validation of siSmad2 and siSmad3 by Western blotting using an antibody detecting both total Smad2 and total Smad3. **(D)** Knockdown of Smad2 significantly increased the mRNA levels of MMP1, IL1b, IL8, ECSCR, and VE-cad. **(E)** Knockdown of Smad3 significantly reduced the mRNA levels of MMP1, IL1b, IL8, ECSCR, and VE-cad. Data represent mean ± SEM (*n* = 3). *p < 0.05; **p < 0.01; ***p < 0.001; and ****p < 0.0001.

To investigate the functional roles of Smad2 and Smad3, two cell lines derived from first trimester placenta, HTR-8/SVneo (41) and Swan 71 (42), were used. Using immunofluorescent staining or western blotting, we confirmed that these cells express epithelial markers, E-cadherin and cytokeratin (CK) 7 or CK8; an EVT marker, HLA-G; but not a fibroblast marker, CD90 (Figure S1). Small interference (si)RNAs targeting Smad2 or Smad3 were transfected into HTR-8/SVneo cells, quantitative real-time PCR showed a significant downregulation of Smad2 mRNA after transfection with Smad2 siRNA (siSmad2, Figure 4A), and of Smad3 mRNA after transfection with siSmad3 (Figure 4B). Furthermore, Western blotting confirmed the down-regulation of Smad2 and Smad3 by their respective siRNAs (Figure 4C). Although siSmad2 significantly increased Smad3 mRNA levels (Figure 4A), it did not increase the protein level of Smad3 (Figure 4C). To investigate the role of Smad2 and Smad3 in the acquisition of an enEVT-like phenotype, we first measured the mRNA levels of several enEVT markers or genes known to play a role in enEVT differentiation (40). Knockdown of Smad2 in HTR-8/SVneo cells resulted in a significant upregulation of vascular endothelial cadherin (VE-cad), matrix metallopeptidase-1 (MMP1), endothelial cell-specific chemotaxis receptor (ECSCR), interleukin-1β (IL1b), and interleukin-8 (IL8) (Figure 4D). In contrast, the mRNA levels of all these genes were downregulated in siSmad3 transfected cells (Figure 4E).

**Figure 5:**
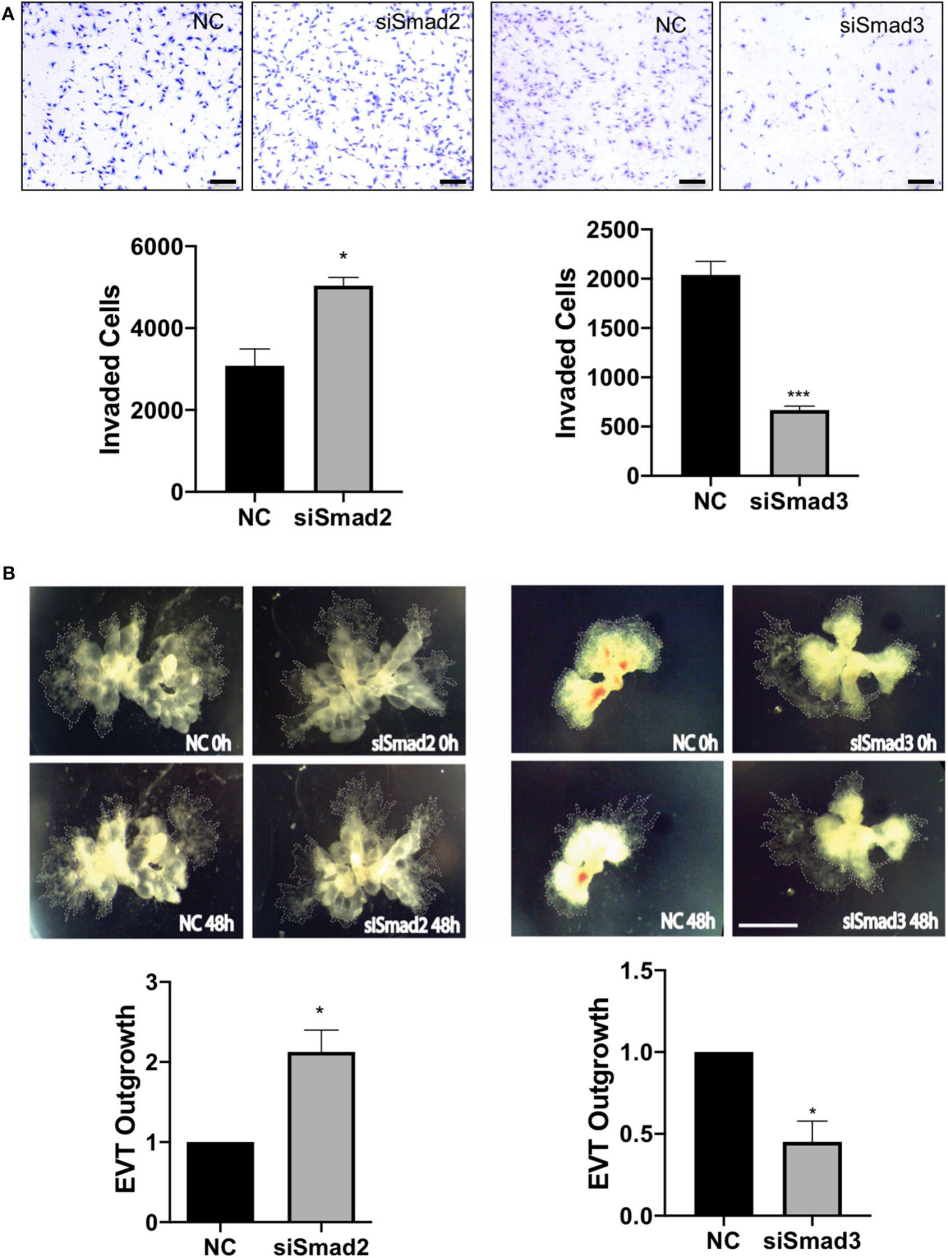
Smad2 suppresses while Smad3 promotes trophoblast invasion and EVT outgrowth. **(A)** HTR-8/SVneo cells transfected with siSmad2 and seeded on matrigel-coated transwells invaded significantly higher than control cells (NC) (left panels). Cells transfected with siSmad3 showed a significant reduction in invasion compared to control cells (right panels). Scale bar = 200 μm. **(B)** Villous explants from 3 first trimester placentas (6, 6.5, and 7.5 week) treated with siSmad2 showed a significantly higher (*p* < 0.05) outgrowth area compared to control tissues (left panels). On the other hand, villous explants from 3 placentas (7, 7.1, and 7.4 week) transfected with siSmad3 had a significant reduction (*p* < 0.05) in EVT outgrowth compared to control explants (right panels). Data represent mean ± SEM (*n* = 3). *p < 0.05 and ***p < 0.001. Representative images from a 6.5 week and a 7 week placental explants are shown for siSmad2 and siSmad3, respectively. The outgrowth area is highlighted by dotted lines. Scale bar = 250 μm.

To further determine the role of Smad2 and Smad3 in the invasive pathway, we measured cell migration and invasion using trophoblast cell lines and EVT outgrowth using first trimester placental explants. Transfection with siSmad2 resulted in a significant increase in the number of cells invaded through the matrigel-coated transwell, while cells transfected with siSmad3 showed a significant reduction in the number of invaded cells, compared to the control (Figure 5A). To confirm the differential effects of Smad2 and Smad3, we transfected with siSmad2, siSmad3, or a non-targeting control into Swan 71 cells. Cell migration was monitored over a period of 48h using the IncuCyte live cell imaging system. Knockdown of Smad2 accelerated, while knockdown of Smad3 suppressed, cell migration (Figure S2). Similarly, in placental explants, knockdown of Smad2 significantly induced the EVT outgrowth, as compared to the control. Whereas, first trimester placental explants treated with siSmad3 showed a smaller EVT outgrowth than the control tissues (Figure 5B).

**Figure 6:**
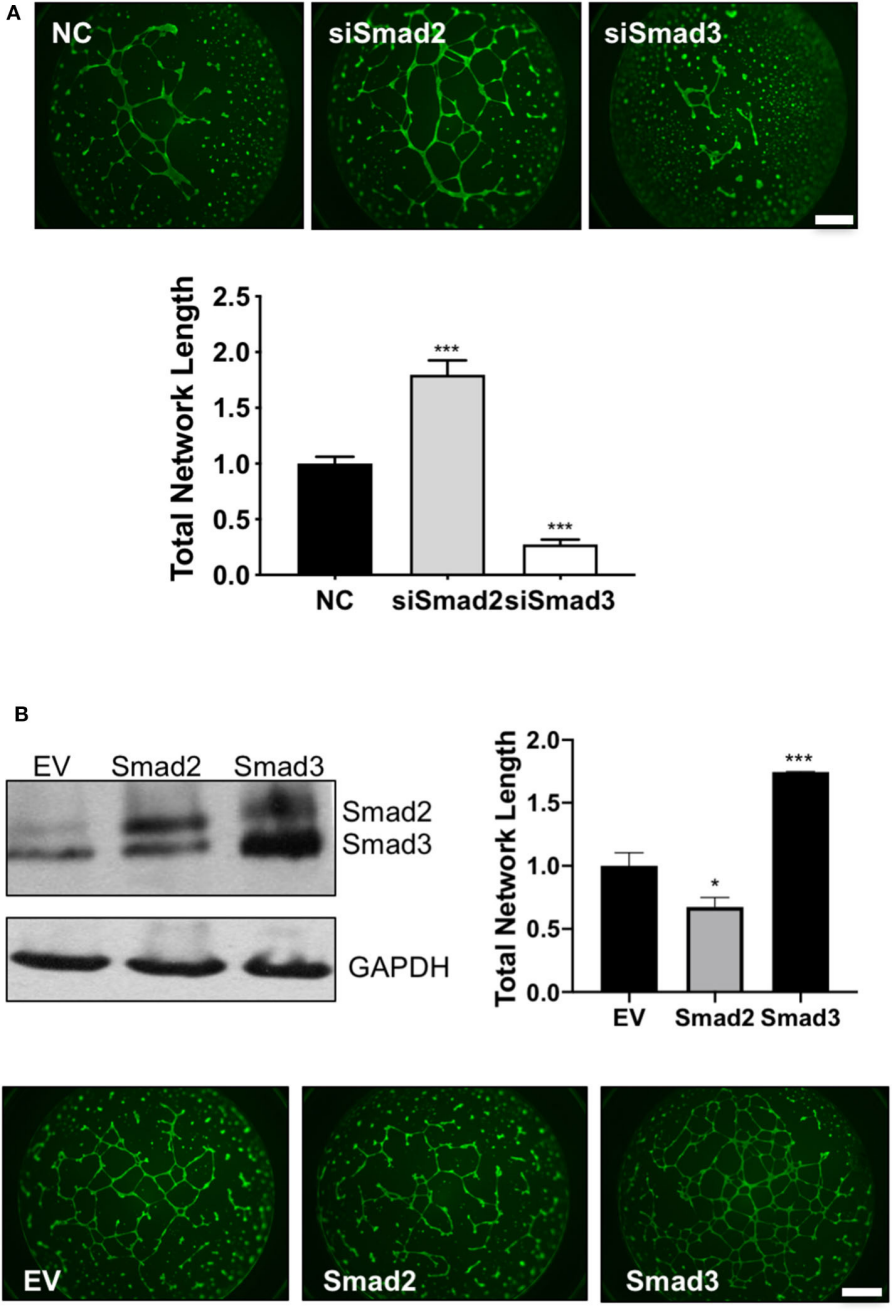
Smad2 suppresses while Smad3 promotes the formation of endothelial-like networks by trophoblasts. **(A)** HTR-8/SVneo cells transfected with siSmad2, siSmad3, or non-targeting control (NC) were seeded on matrigel-coated wells. After 16 h, cells were stained with Calcein AM and total network length was measured. siSmad2 significantly promoted, while siSmad3 significantly suppressed, the ability of trophoblasts to form network structures. **(B)** Overexpression of Smad2 significantly reduced while Smad3 significantly enhanced the total network length compared to empty vector control (EV). HTR-8/SVneo cells were transfected with EV, Smad2-, or Smad3-expressing plasmids. Overexpression of Smad2 and Smad3 was confirmed by Western blotting. Representative network formation and quantitative graphs (mean ± SEM, *n* = 3) were shown. *p < 0.05 and ***p < 0.001. Scale bar = 1 mm.

Finally, endothelial-like network formation assays were performed to further assess the role of Smad2 and Smad3 in the acquisition of enEVT-like phenotype. Trophoblasts transfected with siSmad2 were able to form a larger network, while cells transfected with siSmad3 formed a network with a significantly shorter total length compared to the control (Figure 6A). Conversely, overexpression of Smad2 significantly reduced, while overexpression of Smad3 significantly increased the total network length compared to the empty vector control (Figure 6B). The network formation was also monitored in the IncuCyte. As shown in the movie files and the summary graph at the end of the experiment, Smad2 overexpression suppressed the formation of endothelial-like networks while Smad3 had the opposite effects (Figure S3).

## Discussion

The present study provides the first evidence that Smad2 and Smad3 have distinct roles in EVT invasion and the acquisition of an enEVT-like phenotype. Specifically, we show that Smad2 suppresses, while Smad3 promotes, trophoblast invasion, EVT outgrowth, formation of endothelial-like networks, and expression of enEVT markers or genes known to be involved in the regulation of enEVT function. These findings, together with the opposite expression pattern of pSmad2 and pSmad3 in the placenta, suggest that differentiation along the enEVT pathway is suppressed by Smad2 but promoted by Smad3.

In this study, we showed that the total levels of Smad2 and Smad3 proteins in placentas from different gestational weeks do not change significantly. However, their activation status, reflected by the ratio of the phosphorylated forms to the total levels, varied with gestational age. Specifically, pSmad3/total Smad3 was higher in early gestation, the period of active EVT invasion and spiral artery remodeling, compared to late gestation, while the opposite is true for the pSmad2/total Smad2 ratio. We also found that during early pregnancy, pSmad2 was prominent in the column CTB but lower in HLA-G^+^ EVT cells at the tip while pSmad3 was positively associated with distal HLAG^+^ EVTs. Our findings are consistent with a previous report that pSmad2 is mainly expressed in the proximal and intermediate regions but largely absent in the distal portion of the anchoring villi (45). Another study also reported that in purified EVTs, Smad3 is predominantly found in the nuclei while Smad2 is located in both cytoplasm and nuclei (46). However, the gestational profile of Smad2 is at odds with Xu et al. who found a significant higher total Smad2 in 11–14 weeks than in 6–10 week and term placentas and a low pSmad2 in the third trimester (45). The reason for such discrepancy is not clear but it could possibly be explained by the use of different antibodies and/or different sample size in these studies. Nevertheless, the differential temporal and spatial expression pattern of pSmad2 and pSmad3 supports the notion that they have distinct functions during placental development.

The activity of Smad2 and Smad3 depends on many factors, with the major one being their phosphorylation at the C-termini by type I receptors. In general, Smad2 and Smad3 are phosphorylated by type I receptors activated by TGFβs, Nodal, and Activins, namely activin-receptor like kinase 5 (ALK5), ALK4, and ALK7 (14, 47). All three isoforms of TGF-β and their receptors, ALK-5 and TGFβ-RII, decrease in intensity with gestational age (48). Similarly, Nodal and ALK have higher levels in the first trimester placentas than in the term placenta (34). On the other hand, Activins are detected in the EVTs throughout pregnancy (49). In addition to ligands and receptors, several accessary proteins and inhibitory Smads could also regulate the differential activation of Smad2 and Smad3. For example, Smad Anchor for Receptor Activation may play a role in the differential activation of Smad2 and Smad3 by TGF-β (50). Smad7 blocks only Smad3, but not Smad2, signaling in mesangial cells (51). Finally, crosstalk between Smad2/3 with other signaling pathways also influences their relative activity. ERK has been shown to increase Smad2 signaling (52) but inhibit Smad3 activity (53). Furthermore, AKT has been reported to sequester Smad3, making it unavailable for phosphorylation by type I receptors (54). These pathways can be activated by many cytokines and growth factors, including members of the TGF-β family (55). Therefore, it is likely that the relative activity of Smad2 and Smad3 is regulated by complex signaling networks. How Smad2 and Smad3 phosphorylation is differentially regulated during placental development remains to be investigated.

Previous studies have demonstrated that Smad2 and Smad3 have distinct roles in regulating cell differentiation during development. For example, Smad3 has been identified as a key promoter of neuronal differentiation and cell fate specification, independent of Smad2 (56), while Smad2, but not Smad3, was shown to be indispensable for normal epiblast development (57). In this study, we present several lines of evidence to suggest that Smad2 and Smad3 exert opposite effects on EVT invasion and the acquisition of an enEVT-like phenotype. First, knockdown of Smad2 increased, while knockdown of Smad3 decreased, the level of MMP1, IL1b, IL8, VE-cadherin, and ECSCR. MMP1 is a collagenase important for deep placental invasion and is downregulated in EVTs of PE placentas (58). Both IL1b and IL8 have been reported to promote trophoblast migration and/or invasion (59, 60). VE-cadherin is a marker of enEVT (1) and its upregulation is essential in the trophoblast-mediated endothelial replacement during spiral artery remodeling (61). ECSCR is known to be expressed selectively in endothelial cells and it has been detected in enEVTs that invade the maternal arterioles (62). Second, knockdown of Smad2 promoted, while knockdown of Smad3 suppressed, trophoblast invasion, migration, and the outgrowth of EVTs in placental explants. Finally, we found that Smad2 inhibited the ability of trophoblasts to form endothelial-like networks while Smad3 had the opposite effects. These findings suggest that Smad2 suppresses while Smad3 promotes differentiation of trophoblast toward the invasive enEVT pathway. To confirm this, future studies will determine if epithelial markers, such as such as E-cadherin and integrin α6β4, will be suppressed by Smad3 and induced by Smad2.

Although the role of Smads in enEVT differentiation has not been well-documented, several studies have reported their involvement in regulating trophoblast invasion and gene expression. Specifically, overexpression of Smad2 reduced trophoblast invasion (63) while silencing of Smad2 attenuated the inhibitory effects of TGF-β on trophoblast invasion (64) and VE-cadherin expression (65). It has been reported that silencing of Smad3 inhibited the pro-invasive effects of Activin (26), suggesting that Smad3 promotes trophoblast invasion. However, the same study also shows that a siRNA targeting Smad2 reduced the effect of Activin on trophoblast invasion (26), which is at odds with our results. Future studies will investigate if the relative expression and activation of Smad2 and Smad3 may determine their functions in trophoblasts.

PE is not only the leading cause of maternal and neonatal morbidity and mortality but also has a long-term negative impact on the health of affected mothers and their babies (66). While the etiology of PE is not fully understood, placental, and maternal dysfunction are believed to be major contributors to the development of PE. Specifically, defective EVT development and poor spiral arterial transformation is a precipitating factor in the development of early onset PE (11). Late onset PE is less severe and are often associated with various maternal stressors which may also lead to placental dysfunction (11, 66). In this study, we demonstrated that Smad2 inhibits while Smad3 stimulates trophoblast invasion and acquisition of an enEVT-like phenotype. Interestingly, we found that pSmad3 levels were strongly down-regulated in both early and late onset PE placentas. Although we did not observe a change in pSmad2, a previous study using a larger sample size has reported a significant increase in pSmad2 levels in early-onset PE, as compared to placentas from preterm labors (45). These findings, together with the differential role of Smad2 and Smad3 in trophoblast invasion, expression of enEVT markers, and formation of endothelial-like networks, suggest that inactivation of Smad3 and probably aberrant activation of Smad2 may lead to defective enEVT differentiation and thereby contributing to the development of early onset PE. The significance of Smad3 inactivation in late onset PE is not clear; however, the TGF-β family has been reported to regulate other aspects of placenta functions, such as apoptosis (25, 29). Dysregulation of villous trophoblast apoptosis is also observed in late onset PE (67). The role of Smad3 in this process remains to be investigated.

HTR-8/SVneo is a transformed cell line developed from a first trimester placenta (41) and has been reported to retain some key features of primary trophoblasts (68). This cell line has been used extensively to study the regulation of trophoblast proliferation, migration/invasion, and differentiation with key findings confirmed using primary trophoblasts or placental explants (32, 34, 40, 69–71). In addition, several studies have found that similar to primary trophoblasts, this cell line contains trophoblast progenitor cells that are capable of undergoing self-renewal and differentiation into EVT and STB lineages (72–74). Recently, we found that miR-218-5p promotes HTR-8/SVneo cell invasion, expression of enEVT markers, formation of endothelial-like networks, and their ability to displace endothelial cells. We also confirmed key results from HTR-8/SVneo cells in placental explants and in an *ex vivo* model of spiral artery remodeling (40). These findings suggest that HTR-8/SVneo is a suitable model to study enEVT differentiation. However, it has also been reported that HTR-8/SVneo contains both epithelial and mesenchymal cells as they express low or no E-cadherin and cytokeratin 7 (CK7) but express high levels of vimentin (75, 76). The HTR-8/SVneo cells in our labs express high levels of E-cadherin and CK8 but not a differentiated fibroblast marker, CD-90 (71). It is possible that changes occur to different batches of HTR-8/SVneo cells under different culture conditions and/or after different passages. It is therefore important to confirm key findings from cell lines using primary tissues. Future studies will determine if Smad2 and Smad3 have differential effects on enEVT marker expression in primary trophoblast and/or placental explants, endothelial/trophoblast interaction, and spiral artery remodeling.

In summary, our study provides evidence for distinct and opposite roles of Smad2 and Smad3 in the acquisition of an enEVT-like phenotype and their potential involvement in PE development. From our findings and those reported by others, we postulate that a delicate balance of Smad2 vs. Smad3 activity exists at the anchoring villi that drives the differentiation of enEVTs. However, the mechanisms underlying the differential activation of Smad2 and Smad3 remain unclear. It is most likely that this involves a spatially and temporally regulated mechanism dependent on the availability of ligands, receptors, accessory proteins, and on their cross-talk with other signaling pathways. Our study is the first to explore the differential role of Smad2 and Smad3 in the enEVT pathway. Furthermore, our findings highlight the importance of further characterizing key proteins involved in the regulation of Smad2/3 phosphorylation states across the feto-placental interface in order to better understand these intricate processes essential to healthy pregnancy outcomes.

## Data Availability Statement

The original contributions presented in the study are included in the article/Supplementary Material, further inquiries can be directed to the corresponding author/s.

## Ethics Statement

The studies involving human participants were reviewed and approved by Mount Sinai Hospital Research Ethics Board, Sinai Health System (REB# 02-0061A and 11-0227-E). The patients/participants provided their written informed consent to participate in this study.

## Author Contributions

JB designed and performed most of the experiments, analyzed the data, and drafted the manuscript. CD, YS, JO'B, PL, SQ, and PY conducted some experiments. SL and SM provided guidance for study design and data interpretation. CD, JB, and CP revised the manuscript. CP supervised the study and was involved in experimental design, data analyses, and manuscript writing. All authors contributed to the drafting and/or revision of the manuscript, and approved the submission of the manuscript.

## Conflict of Interest

The authors declare that the research was conducted in the absence of any commercial or financial relationships that could be construed as a potential conflict of interest.
