# Supplementary material for: Differential Role of Smad2 and Smad3 in the Acquisition of an Endovascular Trophoblast-Like Phenotype and Preeclampsia
Source: Front Endocrinol (Lausanne). 2020 Jul 8;11:436. doi: 10.3389/fendo.2020.00436 (PMC7362585; doi:10.3389/fendo.2020.00436)
Supplement: Supplementary file 1 [file Presentation_1.PPTX]

## Slide 1
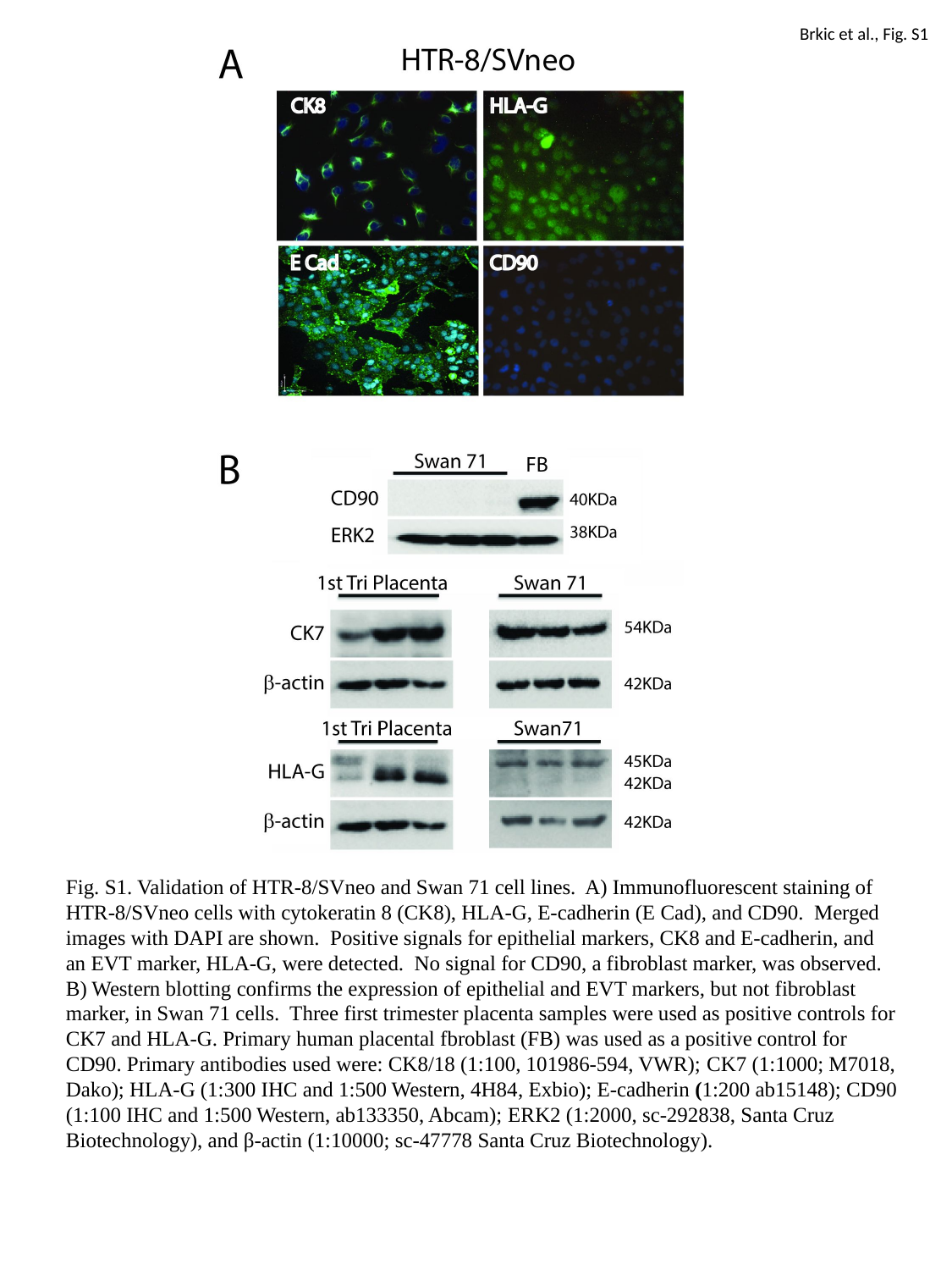

Brkic et al., Fig. S1
Fig. S1. Validation of HTR-8/SVneo and Swan 71 cell lines. A) Immunofluorescent staining of HTR-8/SVneo cells with cytokeratin 8 (CK8), HLA-G, E-cadherin (E Cad), and CD90. Merged images with DAPI are shown. Positive signals for epithelial markers, CK8 and E-cadherin, and an EVT marker, HLA-G, were detected. No signal for CD90, a fibroblast marker, was observed. B) Western blotting confirms the expression of epithelial and EVT markers, but not fibroblast marker, in Swan 71 cells. Three first trimester placenta samples were used as positive controls for CK7 and HLA-G. Primary human placental fbroblast (FB) was used as a positive control for CD90. Primary antibodies used were: CK8/18 (1:100, 101986-594, VWR); CK7 (1:1000; M7018, Dako); HLA-G (1:300 IHC and 1:500 Western, 4H84, Exbio); E-cadherin (1:200 ab15148); CD90 (1:100 IHC and 1:500 Western, ab133350, Abcam); ERK2 (1:2000, sc-292838, Santa Cruz Biotechnology), and β-actin (1:10000; sc-47778 Santa Cruz Biotechnology).
