# Supplementary material for: Differential Role of Smad2 and Smad3 in the Acquisition of an Endovascular Trophoblast-Like Phenotype and Preeclampsia
Source: Front Endocrinol (Lausanne). 2020 Jul 8;11:436. doi: 10.3389/fendo.2020.00436 (PMC7362585; doi:10.3389/fendo.2020.00436)
Supplement: Supplementary file 2 [file Presentation_2.PPTX]

## Slide 1
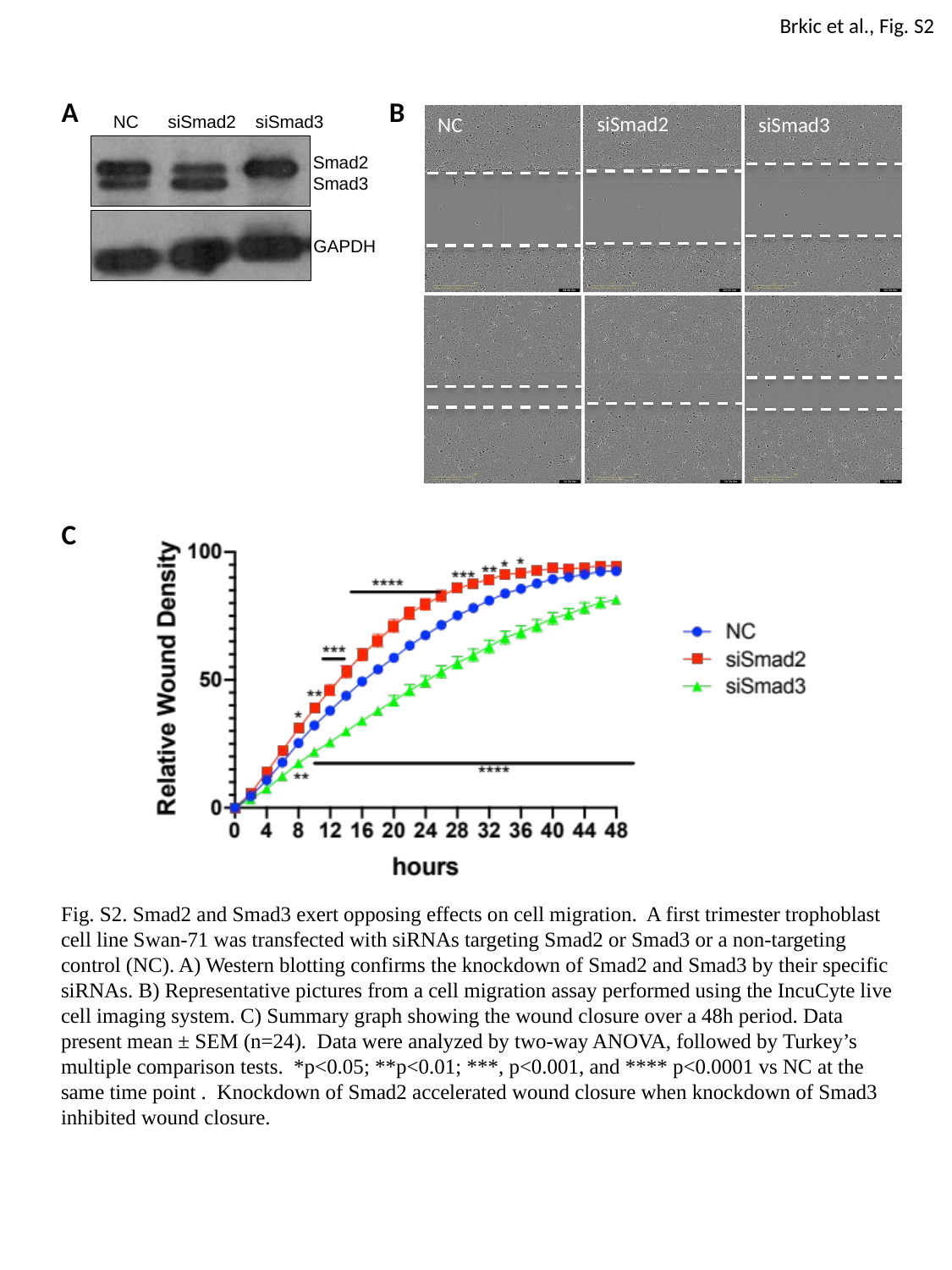

Brkic et al., Fig. S2
A
B
NC siSmad2 siSmad3
siSmad2
NC
siSmad3
Smad2
Smad3
GAPDH
C
Fig. S2. Smad2 and Smad3 exert opposing effects on cell migration. A first trimester trophoblast cell line Swan-71 was transfected with siRNAs targeting Smad2 or Smad3 or a non-targeting control (NC). A) Western blotting confirms the knockdown of Smad2 and Smad3 by their specific siRNAs. B) Representative pictures from a cell migration assay performed using the IncuCyte live cell imaging system. C) Summary graph showing the wound closure over a 48h period. Data present mean ± SEM (n=24). Data were analyzed by two-way ANOVA, followed by Turkey’s multiple comparison tests. *p<0.05; **p<0.01; ***, p<0.001, and **** p<0.0001 vs NC at the same time point . Knockdown of Smad2 accelerated wound closure when knockdown of Smad3 inhibited wound closure.
