# Supplementary material for: Differential Role of Smad2 and Smad3 in the Acquisition of an Endovascular Trophoblast-Like Phenotype and Preeclampsia
Source: Front Endocrinol (Lausanne). 2020 Jul 8;11:436. doi: 10.3389/fendo.2020.00436 (PMC7362585; doi:10.3389/fendo.2020.00436)
Supplement: Supplementary file 3 [file Presentation_3.PPTX]

## Slide 1
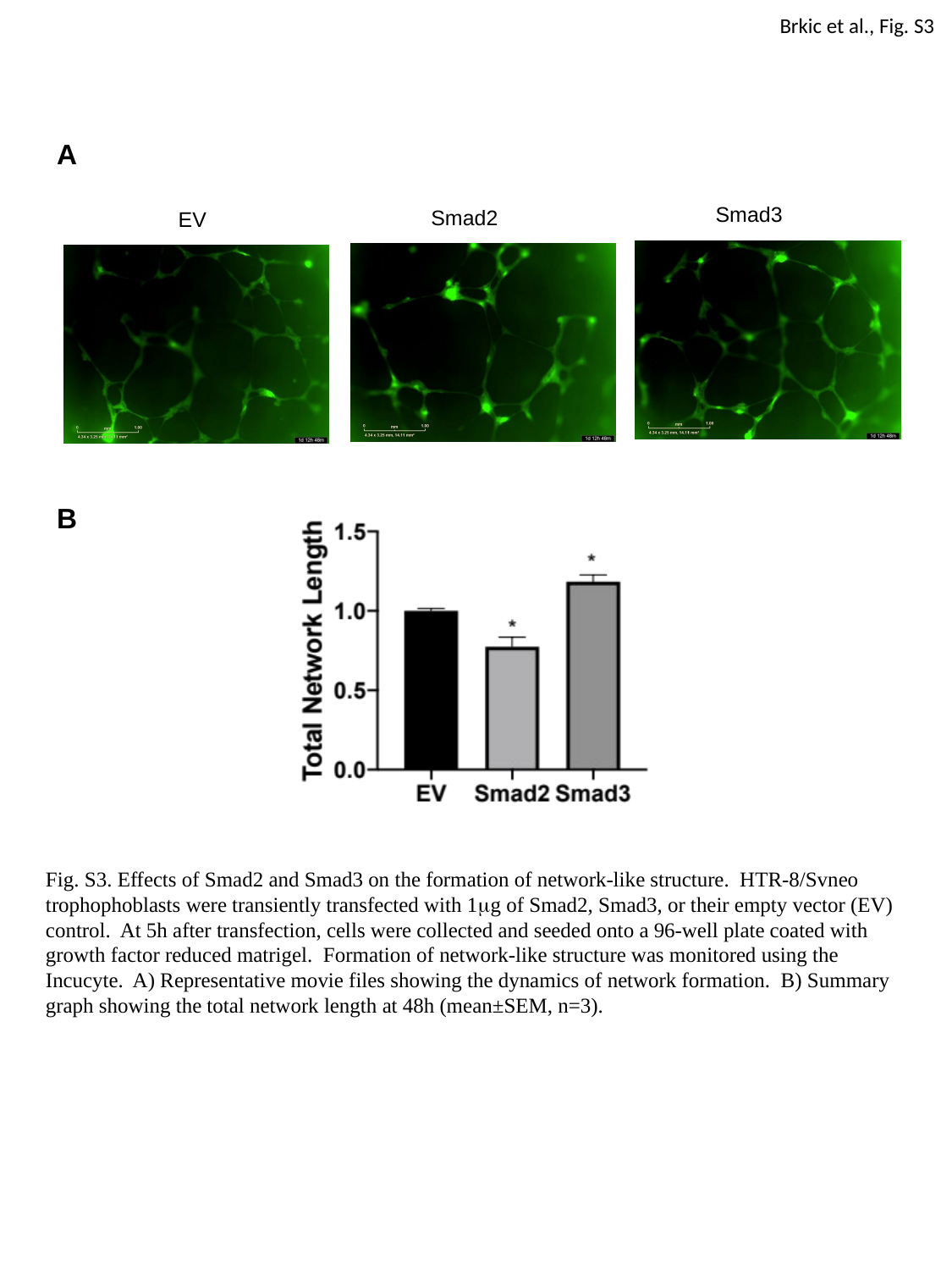

Brkic et al., Fig. S3
A
Smad3
Smad2
EV
B
Fig. S3. Effects of Smad2 and Smad3 on the formation of network-like structure. HTR-8/Svneo trophophoblasts were transiently transfected with 1mg of Smad2, Smad3, or their empty vector (EV) control. At 5h after transfection, cells were collected and seeded onto a 96-well plate coated with growth factor reduced matrigel. Formation of network-like structure was monitored using the Incucyte. A) Representative movie files showing the dynamics of network formation. B) Summary graph showing the total network length at 48h (mean±SEM, n=3).
